# Physical activity and transitioning to retirement: evidence from the China health and retirement longitudinal study

**DOI:** 10.1186/s12889-023-16870-7

**Published:** 2023-10-06

**Authors:** Huiyan Fang, Zihui Xiong, Yilin Li, Wenhui Cui, Ziping Cheng, Ji Xiang, Ting Ye

**Affiliations:** 1 School of Medicine and Health Management, Tongji Medical College, Huazhong University of Science and Technology, Wuhan, China; 2 Department of Education, Research Center for Rural Health Service, Key Research Institute of Humanities & Social Sciences of Hubei Provincial, Wuhan, China

**Keywords:** Physical activity, Retirement, Latent class growth analysis

## Abstract

**Background:**

The impact of retirement on physical activity among older individuals remains ambiguous. This study aims to investigate the influence of retirement on physical activity and delineate the trajectories of physical activity changes during the retirement transition among elderly Chinese residents. Additionally, we endeavor to examine the factors that contribute to each trajectory.

**Methods:**

This longitudinal cohort study used data from four surveys of the China Health and Retirement Longitudinal Study and included a sample of 428 individuals who underwent formal retirement and provided information on physical activity. We employed generalized estimating equation to explore the impact of the retirement transition on physical activity among Chinese older adults. Latent class growth analysis was used to identify distinct trajectories of physical activity, and binary logistic regression was performed to identify pre-retirement factors influencing changes in physical activity.

**Results:**

Our findings indicate that retirement can lead to a decline in physical activity among older Chinese residents (OR = 0.85, 95%CI 0.75 ~ 0.97). We identified three distinct trajectories of physical activity during the retirement transition: Trajectory 1 – “sustained low level of physical activity” (7.94%); Trajectory 2 – “middle level of physical activity with gradual decline” (69.16%); Trajectory 3 – “sustained high level of physical activity with significant fluctuations” (22.90%). Furthermore, we discovered that individuals in the “middle level of physical activity and gradual decline” trajectory were more likely to have an annual income exceeding 40,000 yuan (OR = 9.69, 95%CI 1.12 ~ 83.63), reside in urban areas (OR = 2.27, 95%CI 1.14 ~ 4.52), and have a fondness for playing Mahjong (OR = 2.42, 95%CI 1.18 ~ 5.00) compared to those in the “sustained high level of physical activity with significant fluctuations” trajectory. Additionally, having an annual income exceeding 40,000 yuan (OR = 19.67, 95%CI 1.30 ~ 298.61) predicted membership in the “sustained low level of physical activity” trajectory when compared to the “sustained high level of physical activity with significant fluctuations” trajectory.

**Conclusion:**

Retirement represents a substantial milestone in the life course and is associated with notable alterations in physical activity patterns. Among older Chinese residents, the trajectories of physical activity during the retirement transition exhibit diverse paths and are influenced by pre-retirement factors, including annual income, residential location, and hobbies. The findings of this study have important implications for the formulation of policies aimed at promoting healthy aging among individuals approaching retirement age.

## Background

Aging is a growing concern worldwide due to the rise in life expectancy [1], and this issue is particularly prominent in China. By 2050, nearly one-fifth of China’s total population is projected to be over the age of 65, resulting in one of the highest proportions of elderly people globally [2]. This demographic shift poses major challenges, including negative impacts on economic growth [3], an increase of chronic non-communicable diseases (CNCDs) [4], and a substantial increase in health costs [5]. Physical activity, as part of a healthy lifestyle, is widely considered a crucial component of healthy aging [6].

Numerous studies have demonstrated the multiple health benefits of physical activity, especially for older adults [7]. Regular physical activity can help reduce the incidence of CNCDs [8], such as coronary heart disease [9] and stroke [10], diabetes [4], hypertension, colon cancer, breast cancer, and depression [11]. There is also a positive association between physical activity levels and health-related quality of life [12]. Thus, it is essential to identify opportunities to promote physical activity among older adults to support healthy aging.

Retirement transition is a significant life event marked by changes in work status, social networks, income, and lifestyles. Previous research has explored the changes in various health behaviors during the retirement transition period, such as smoking [13], alcohol consumption [14], sleep patterns [15], self-rated health [16]. Another noteworthy health behavior that undergoes changes during the retirement transition period is physical activity. The relationship between retirement and physical activity has been extensively studied, but with inconsistent findings [17, 18]. Some researchers have reported an increase in physical activity during the retirement transition [19–22], while others have observed a significant decrease [23, 24]. A considerable number of studies have presented mixed results, suggesting that physical activity may initially increase shortly after retirement (e.g., 3 to 12 months) and then gradually decline [25–28]. There may be considerable individual differences in changes in physical activity during the transition to retirement, depending on factors such as occupational categories, living environments, and life status [29, 30]. A review of existing literature reveals no consensus on how physical activity changes during retirement. It is possible that these different results are due to gaps in research methodology, age of the study participants, occupational background and other factors.

Previous studies investigating retirement behavior patterns have adopted longitudinal methods, such as latent trajectory analysis, to explore multiple domains, including sleep [15], self-rated health [31], smoking [13], excessive alcohol consumption [32]. Latent Class Growth Analysis (LCGA), a data-driven approach, identifies groups with similar developmental trajectories over time [33]. However, there are limited studies using LCGA to analyze changes in physical activity levels during the retirement transition, with most literature relying on linearity test methods [27, 34], which can only make use of a relatively small amount of data and may not give a fuller picture of the actual situation.

Furthermore, due to cultural and environmental differences, the physical activity patterns of older Chinese residents may differ significantly from those in Western countries [35, 36]. The extant body of research demonstrates that factors influencing physical activity among elderly residents in China diverge from those observed among elderly populations in other nations. Chief among these distinctions is the salient role played by the household registration (hukou) system. Each Chinese citizen is classified into an agricultural or non-agricultural household registration system. By design, two-fold stratification structure was linked to unequal access to social benefit, and that non-agricultural residents tend to receive benefits that are not available to their rural counterparts Given the pronounced urban-rural disparities in China, elderly individuals holding rural household registrations are more prone to facing suboptimal economic conditions, necessitating their sustained engagement in gainful labor. Moreover, the deeply ingrained familial ethos within Chinese society often compels elderly individuals to assume responsibilities for intergenerational caregiving, thereby exerting a substantial influence on their physical activity patterns. In addition to the examined factors, notwithstanding prior investigations utilizing LCGA to explore the dynamics of physical activity among elderly residents pre- and post-retirement in countries such as the United States and the United Kingdom, an analogous application of this methodology to scrutinize the physical activity patterns of China’s elderly populace remains conspicuously absent, necessitating further research. [37, 38]

To provide new insights into changes in physical activity during the retirement transition, we utilized data from all four phases of the China Health and Retirement Longitudinal Study (CHARLS) survey. The objectives of this study were to (1) examine the effect of retirement transition on physical activity among older Chinese residents, (2) identify trajectories of physical activity change during the retirement transition, and (3) investigate the factors influencing each trajectory.

## Methods

### Design and study population

CHARLS is a nationally representative longitudinal survey of individuals aged 45 years and older in China. The survey, conducted in four waves (2011, 2013, 2015, and 2018), assesses the social, economic, and health status of community residents [39].

**Fig. 1:**
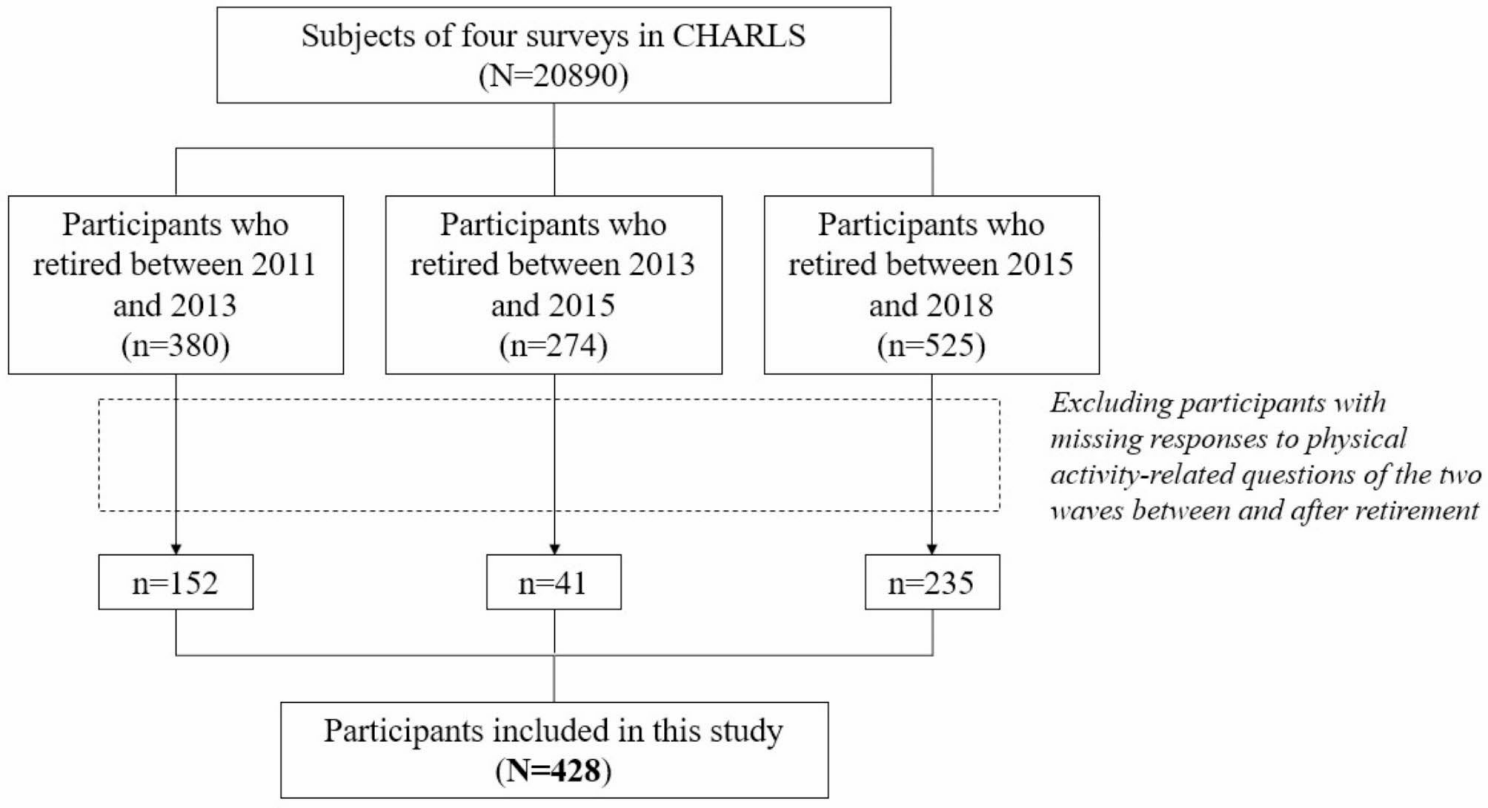
Selection process of research subjects

We selected individuals who reported having formally retired in all four waves of CHARLS. We restructured the longitudinal dataset to center around retirement transition. For example, the wave before retirement is noted as wave-1, wave-2, wave-3 and the wave after retirement is noted as wave + 1, wave + 2, wave + 3. As only half of the participants answered the physical activity-related questions in the first three surveys, we only included participants who provided physical activity-related data for both the pre-retirement (wave-1) and post-retirement (wave + 1) wave. In the end, a total of 428 individuals were included as subjects for this study. Detail procedure of selection is shown in Fig. 1.

### Assessment of physical activity

Questions DA051 to DA055 assess the duration and frequency of vigorous, moderate, and light physical activity performed in a typical week. Question DA051 asks:“ During a usual week, did you do any vigorous physical activity or moderate physical activity, or light physical activity for at least 10 minutes continuously?” Question DA052 asks:“ During a usual week, how many days did you do vigorous physical activity or moderate physical activity, or light physical activity for at least 10 minutes?” Question DA053-DA055 asks:“ How much time did you usually spend doing vigorous physical activity or moderate physical activity, or light physical activity on one of those days?”

Therefore, we obtained data on whether the study subjects engaged in physical activity from DA051. Data on the number of days per week the study subjects engaged in physical activity were obtained from DA052. Additionally, data on the duration of specific types of physical activity performed by the study subjects on the days they engaged in physical activity were obtained from DA053-DA055.

As CHARLS only provides broad time intervals for each type of physical activity, we converted the responses into average time intervals to more accurately measure the duration of physical activity [40]. If the reported duration of physical activity is more than 10 min but less than 30 min, we define it as 20 min of physical activity. If the reported duration is more than 30 min but less than 2 h, we define it as 75 min of physical activity. If the reported duration is 2 h but less than 4 h, we define it as 180 min of physical activity. If the reported duration is more than 4 h, we define it as 300 min of physical activity.

Due to the varying significance of different types of physical activity for overall health, we aim to handle these differences more appropriately by adopting the International Physical Activity Questionnaire (IPAQ) criteria to calculate the intensities of physical exertion for different types of physical activity, using metabolic equivalent (MET) as the unit of measurement. We calculated MET scores for each respondent based on the time spent on various physical activities each week [41]. Consequently, we calculated MET multipliers as follows: MET score = 8.0*the time of vigorous physical activity in a week + 4.0*the time of moderate physical activity in a week + 3.3*the time of light physical activity in a week.

### Covariates

The data of the covariates included in this study were obtained from wave-1. Our study included several important sociodemographic variables as covariates: age, gender, education level, marital status, and hukou, a unique Chinese form of citizenship, divided into agricultural and non-agricultural hukou.

Our study also incorporated socioeconomic factors, including annual income. Due to the significant impact of living environment on the physical activity of older adults, and the notable urban-rural divide in China with substantial differences in social environment and infrastructure between urban and rural areas, our study included the factor of residential area, categorized as urban and rural. Additionally, this study incorporated health-related factors, such as Body Mass Index (BMI) and the presence of chronic diseases, with BMI being a continuous variable. Furthermore, since this study focused on Chinese elderly residents, the factor of playing Mahjong was included. Mahjong is a unique social activity in China that is widely popular among the elderly population and involves various cognitive abilities such as calculation and attention. The activity of mahjong entails prolonged seated engagement for residents, involving cognitive computations, predominantly engaging the upper extremities in mild exertion. A single session of mahjong typically endures several hours, thus individuals with a proclivity for sustained mahjong participation may find themselves precluded from allocating time to engage in physical exercise. Moreover, the protracted periods of sedentary behavior associated with extended mahjong sessions can give rise to discernible ramifications for physical well-being [42].

### Statistical analysis

The statistical analysis is conducted in three steps.

**Fig. 2:**
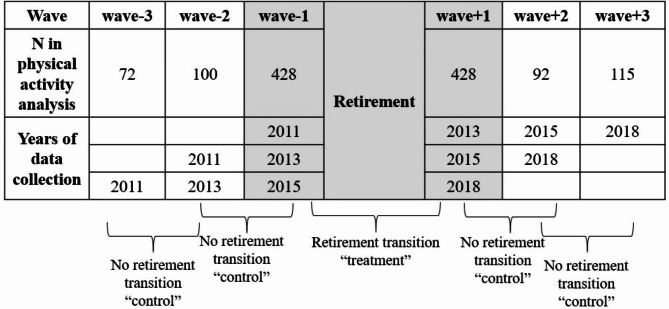
Number of CHARLS participants at each possible survey wave and illustration of the survey wave pairs included in the pseudo trial

First, we examined the effect of retirement transition on physical activity by using a design that treats longitudinal observational data as a series of non-randomized pseudo-trials, simulating the selection process typically seen in clinical trials [13, 43]. The surveys in which participants retired between them were considered the “treatment” group, while surveys in which participants were either non-retirees or retirees at both survey waves were designated as the “control” group. Figure 2 provides detailed information. We compared the MET score between the treatment and the control groups. We used generalized estimating equations (GEE) to calculate odds ratios (OR) and 95% confidence intervals (95% CI) for the score of MET per week in both groups. This analysis was performed using SPSS 26.

Second, we employed latent class growth analysis (LCGA) to identify different types of physical activity trajectories during retirement transition. LCGA, which is considered of advantage in identify clusters of individuals that follow a similar pattern of change on a variable over time [44–46], was used in this current study to explore the latent groups for longitudinal physical activity. Due to the international recommendation of at least 150 min of moderate-intensity physical activity per week for older adults, our study utilized 150 min of moderate-intensity physical activity, corresponding to 600 MET-minutes, as the threshold [47, 48]. The dependent variable was transformed into a binary variable with values of 0–1 for further research. The standards for the best-fitting trajectory model are as follows [33, 49]: (1) Bayesian information criterion (BIC) and Akaike’s Information Criterion (AIC), smaller values means a better-fitting trajectory model; (2) Average posterior probabilities (AvePP), the bigger AvePP means a better-fitting trajectory model; (3) The p-value, p-values for each group should exhibit statistical significance, which means they should be equal to or less than 0.05; (4) Smallest group member, the smallest group member should be more than 5% of the total study population.

Third, we examined which pre-retirement factors predicts the membership of each trajectory by using binary logistic regression. The likelihood of membership in each trajectory is measured by OR and 95% CI. Steps two and three were performed using Stata 16.

## Results

### Test of sample representativeness

In the four waves of the CHARLS survey, a total of 1,179 individuals reported experiencing formal retirement during this period. Among them, 428 individuals were included in our study. To ensure that our study sample represents Chinese retired residents to a certain extent, we conducted a Pearson chi-square test to examine the representativeness of the sample. To be more specific, the overall population is 1,179 individuals who experienced retirement between 2011 and 2018, and the sample consisted of 428 individuals who experienced retirement and provided physical activity data. Test factors included age (15% vs. 16% 65 years old and over), gender (47% vs. 45% female), education level (67% vs. 68% secondary or lower), marital status (93% vs. 91% with partnership), and hukou (35% vs. 34% agricultural hukou), which are fundamental sociodemographic characteristics. Given the profound influence of health status on the level of physical activity among the elderly, in addition to assessing the aforementioned fundamental attributes, we have also incorporated the variable of chronic illness status into our analysis (74%vs. 76% suffering from chronic diseases). The results of the chi-square test indicate that there were no significant differences (p-value > 0.05) between the sample and the population for all tested factors. This suggests that the sample is representative of the overall population.

### The basic overview of physical activity

**Fig. 3:**
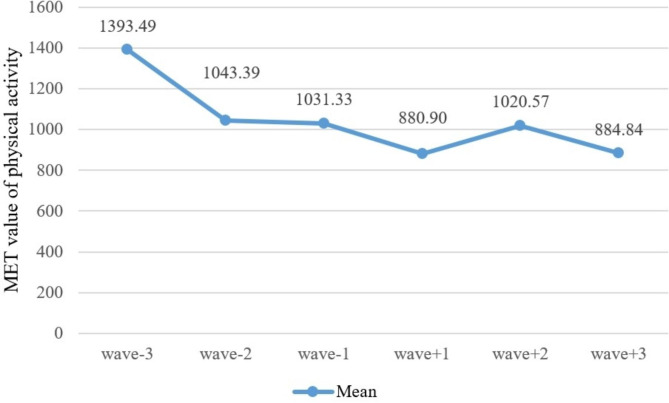
Mean of each wave’s MET value of physical activity

Figure 3 illustrates the specific values and variations in the average MET values of physical activity across all six survey waves in our study. It can be observed that the average MET values gradually decline over the six periods, with a slight increase in wave + 2 but an overall downward trend.

### Pseudo-trial for physical activity

In the pseudo-trial on physical activity, there were 428 participants in the treatment group (i.e. retirement transition) and 379 in the control group (i.e. no retirement transition). Due to the original MET values being predominantly below 1000, with a small portion reaching close to 4000, the raw data exhibited a skewed distribution. To ensure a better fit of the GEE model, we performed a natural logarithmic transformation on the data. This transformation resulted in the data approximating a normal distribution, which was then used for analysis. Compared with the control group, retirement transition was associated with a lower likelihood of physical activity (OR = 0.852, 95% CI = 0.749 ~ 0.968; detailed information in Table 1).

**Table 1:** Pseudo-trials on the effect of retirement on change in physical activity

	Outcome: MET score of physical activity after a natural logarithm transformation				
	Number of participants	Number of paired observations	Mean number of paired observations/person	OR	95% CI
No retirement transition¹ ‘control’	379	299	0.8	1(ref)	
Retirement transition² ‘treatment’	428	428	1	0.852*	0.749 ~ 0.968

Note: ¹ Refers to time periods before retirement (wave-3 to wave-2; wave-2 to wave-1) or after retirement (wave + 1 to wave + 2; wave + 2 to wave + 3)

² Refers to time period of retirement transition (wave-1 to wave + 1)

*: p < 0.05

### Latent classes for physical activity

**Fig. 4:**
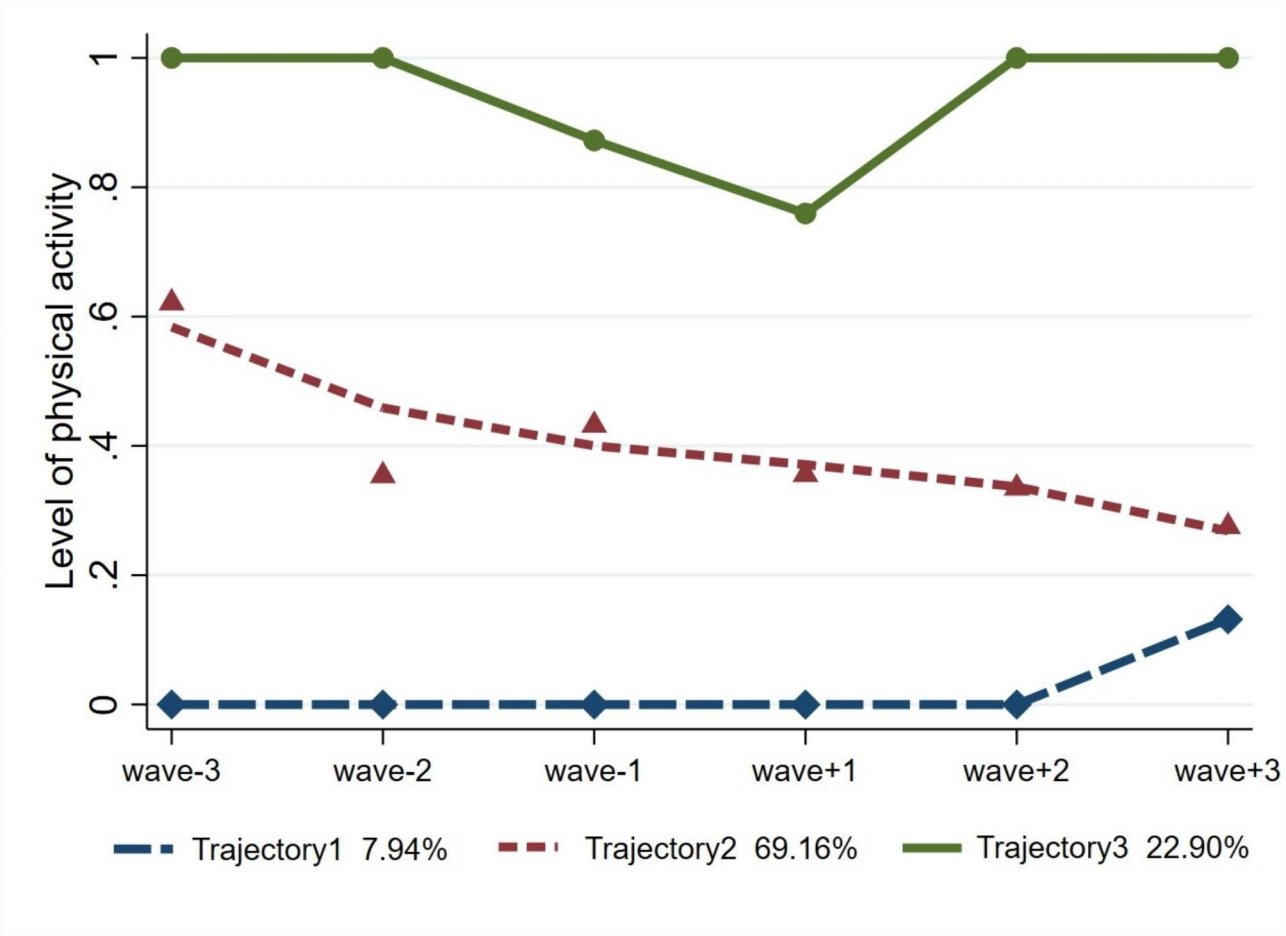
Three different trajectories of physical activity during retirement transition

To identify different trajectories of physical activity changes in Chinese people during the retirement transition, we tested two-, three-, four-, and five-class models. After iterating through various models, the three-class model was found to best fit the data. To ensure the most suitable order for the four-class model, detailed fit indices are presented in Table 2. The “232” model is the optimal choice for further research. The results of the “232” model are shown in Fig. 4, revealing three distinct trajectories: Trajectory 1- “sustained low level of physical activity” (7.94%); Trajectory 2- “middle level of physical activity with gradual decline” (69.16%); Trajectory 3- “sustained high level of physical activity with significant fluctuations” (22.90%).

**Table 2:** Fit indices of different class models of physical activity among the total study population

Order of three-classed model	BIC	AIC	Average posterior probabilities	Smallest group member (%)
223	827.23	802.87	0.75	7.72
232*	**827.17**	**802.81**	**0.75**	**7.94**
233	830.03	803.65	0.75	7.83
333	832.25	803.84	0.70	13.28

Notes: *: model which is chosen for further research

BIC: Bayesian information criterion; AIC: Akaike’s Information Criterion

### Pre-retirement factors predicted group membership

In the subsequent analysis, we investigated the association between group membership and pre-retirement (wave-1) characteristics. The pre-retirement characteristics of each member in three trajectories are presented in Table 3. The results of binary logistic regression are presented in Table 4. Those in the “middle level of physical activity with gradual decline” trajectory were more likely to have an annual income exceeding 40,000 yuan, and reside in urban areas, and have a fondness for playing Mahjong when compared with the “sustained high level of physical activity with significant fluctuations”. We found having an annual income exceeding 40,000 yuan predicts membership of the “sustained low level of physical activity” trajectory when compared with the “sustained high level of physical activity with significant fluctuations”.

**Table 3:** Pre-retirement characteristics of each trajectory

Characteristics	Trajectory 1 (N = 34)			Trajectory 2 (N = 296)			Trajectory 3 (N = 98)	
	n	%		n	%		n	%
**Age**								
< 50	6	17.65		72	24.32		20	20.41
50 ~ 60	18	52.94		139	46.96		50	51.02
> 60	10	29.41		85	28.72		28	28.57
**Gender**								
Women	17	50.00		148	50.00		39	39.80
Men	17	50.00		148	50.00		59	60.20
**Education Level**								
Secondary or lower	19	55.88		195	65.88		74	75.51
High school or higher	15	44.12		101	34.12		24	24.49
**Marital Status**								
Without partnership	4	11.76		21	7.09		2	2.04
With partnership	30	88.24		275	92.91		96	97.96
**Hukou**								
Agricultural hukou	9	26.47		93	31.42		50	51.02
Non-agricultural hukou	24	70.59		178	60.14		46	46.94
missing	1	2.94		25	8.44		2	2.04
**Annual Income**								
< 10,000	20	58.82		166	56.08		57	58.16
10,000 ~ 20,000	2	5.88		37	12.50		19	19.39
20,000 ~ 30,000	1	2.94		30	10.14		9	9.18
30,000 ~ 40,000	4	11.76		28	9.46		8	8.16
≥ 40,000	6	17.65		31	10.47		4	4.08
missing	1	2.94		4	1.35		1	1.02
**Residential Area**								
Rural	6	17.65		64	21.62		37	37.76
Urban	28	82.35		232	78.38		61	62.24
**BMI**								
mean	25.10	/		26.70	/		24.10	/
missing	7	/		86	/		14	/
**Chronic Diseases**								
No	10	29.41		67	22.64		26	26.53
Yes	24	70.59		229	77.36		72	73.47
**Mahjong Playing**								
No	27	79.41		215	72.64		82	83.67
Yes	7	20.59		81	27.36		16	16.33

**Table 4:** Pre-retirement (wave-1) factors predicted group membership

Characteristics	Trajectory 1 vs. Trajectory 3			Trajectory 2 vs. Trajectory 3	
	OR	95% CI		OR	95% CI
**Age**					
< 50 (ref)					
50 ~ 60	1.07	0.21 ~ 5.41		1.06	0.47 ~ 2.40
> 60	1.85	0.28 ~ 12.16		1.59	0.60 ~ 4.22
**Gender**					
Women (ref)					
Men	0.32	0.09 ~ 1.19		0.71	0.36 ~ 1.38
**Education Level**					
Secondary or lower (ref)					
High school or higher	1.44	0.50 ~ 4.12		1.20	0.68 ~ 2.14
**Marital Status**					
Without partnership (ref)					
With partnership	0.14	0.02 ~ 1.12		0.45	0.09 ~ 2.20
**Hukou**					
Agricultural hukou (ref)					
Non-agricultural hukou	1.07	0.28 ~ 3.99		1.05	0.55 ~ 2.01
**Annual Income**					
< 10,000 (ref)					
10,000 ~ 20,000	0.36	0.06 ~ 2.23		0.76	0.34 ~ 1.68
20,000 ~ 30,000	1	/		1.21	0.43 ~ 3.40
30,000 ~ 40,000	2.40	0.40 ~ 14.27		1.09	0.38 ~ 3.10
≥ 40,000	19.67*	1.30 ~ 298.61		9.69*	1.12 ~ 83.63
**Residential Area**					
Rural (ref)					
Urban	1.86	0.44 ~ 7.81		2.27*	1.14 ~ 4.52
**BMI**					
mean	1.09	0.94 ~ 1.26		1.02	0.94 ~ 1.10
**Chronic Diseases**					
No (ref)					
Yes	0.62	0.16 ~ 2.46		1.00	0.53 ~ 1.89
**Mahjong Playing**					
No (ref)					
Yes	1.16	0.24 ~ 5.60		2.42*	1.18 ~ 5.00

Notes: *: p ≤ 0.05

## Discussion

In our large longitudinal cohort study on Chinese older people who entered into statutory retirement, during a certain period before and after retirement, the physical activity levels of older adults tend to gradually decline. However, a transient surge in average physical activity levels is discernible during the immediate post-retirement period, succeeded by a subsequent gradual decline The transition phase to retirement was associated with a lower level of physical activity compared with times before and after retirement transition. Three trajectories of physical activity were found: Trajectory 1- “sustained low level of physical activity” (7.94%); Trajectory 2- “middle level of physical activity with gradual decline” (69.16%); Trajectory 3- “sustained high level of physical activity with significant fluctuations” (22.90%). Those in the “middle level of physical activity with gradual decline” trajectory were more likely to have an annual income exceeding 40,000 yuan, and reside in urban areas, and have a fondness for playing Mahjong when compared with the “sustained high level of physical activity with significant fluctuations”. Also, we found having an annual income exceeding 40,000 yuan predicts membership of the “sustained low level of physical activity” trajectory when compared with the “sustained high level of physical activity with significant fluctuations”.

According to the World Health Organization’s recommendation of a minimum of 150 min of moderate-intensity physical activity per week for older individuals [48], which corresponds to at least 600 MET per week, our findings suggest that the average level of physical activity of older Chinese individuals during the retirement transition is basically up to international standards, indicating a healthy lifestyle. However, with the increase in age, there is a noticeable decline in the physical activity levels of Chinese older adults. This trend is not conducive to maintaining good health. In this longitudinal study from China, we observed a general decrease in physical activity during the retirement transition, same with before and after this period.

However, it is worth noting that during the period from wave + 1 to wave + 2, there was a noticeable increase in physical activity levels, although it continued to decline after wave + 2. Consistent with previous related studies, this increase may be attributed to a temporary rise in physical activity levels among older adults shortly after retirement, with a duration typically ranging from 3 to 12 months [10, 24, 26, 27, 40, 50]. The increase in physical activity during this period may be due to higher psychological well-being and increased leisure time immediately after retirement, leading to a significant increase in light-intensity physical activity. However, as years pass after retirement and with advancing age and declining physical function, physical activity levels among older adults gradually decrease [28]. Most previous studies on the association between retirement and physical activity have utilized cross-sectional data or longitudinal data with relatively short intervals. In contrast, our study utilized data spanning eight years and six waves, allowing us to observe a continuous fluctuation pattern of physical activity that initially declines, then increases, and subsequently declines again. This extended duration of data collection in our study provides a more comprehensive understanding compared to previous studies that primarily focused on singular outcomes of either increase or decrease in physical activity.

To investigate the impact of retirement on older individuals’ physical activity, we first employed GEE to examine whether retirement transition influenced the physical activity of this demographic. Some longitudinal studies have reported decreasing physical activity during retirement transition [23, 24, 26, 27, 40]. Based on the responses of the question asking the purpose of each kind of intensity physical activity, it can be observed that for high-intensity physical activities, 88.42%, 84.82%, and 82.96% of residents respectively answered that they engaged in such activities for work-related reasons. For moderate-intensity physical activities, 66.66%, 60.84%, and 54.29% of residents respectively answered for work-related reasons. For light-intensity physical activities, 51.92%, 49.16%, and 41.91% of residents respectively answered for work-related reasons. It is evident that a significant portion of physical activities among Chinese older adults is driven by work-related needs, particularly for high-intensity physical activities which have the greatest impact on MET values. More than 80% of residents engage in these activities for work purposes. Therefore, although Chinese older adults meet the recommended international activity levels, it does not necessarily imply that they have healthy exercise habits. After retirement, as they cease working, they lose the physical activities that were part of their job responsibilities. Consequently, this leads to a gradual decline in physical activity levels before and after retirement [23].

Subsequently, we employed LCGA to explore various types of physical activity changes in older individuals undergoing the retirement transition. We found that the development trajectories of physical activity among Chinese elderly individuals could be classified into three categories: sustained low, middle level with gradual decline, sustained high level with significant fluctuations. Almost all older individuals experienced decreasing in physical activity during the retirement transition. Similar conclusions have been reached in related studies, suggesting that physical activity changes during the retirement transition vary due to differences in individual experiences [29, 30]. For the group of individuals with sustained high levels of physical activity with significant fluctuations, we can observe a significant decline in their activity levels during the retirement transition period. However, after a certain period of transition, their physical activity levels gradually increase and return to the high pre-retirement levels. Taking into account that over 80% of respondents indicated that the purpose of engaging in vigorous physical activity was work-related, and considering the substantial contribution of vigorous physical activity to the overall MET values, we can speculate that the change in their work status during the retirement transition period results in a loss of the physical activities associated with work [51]. This leads to an overall decline in the level of physical activity. However, these individuals are relatively conscious of exercise or have established exercise habits. Once the transition period is over and their lives return to a relatively stable state, they are able to resume regular exercise. Consequently, the overall physical activity level can regain a stable and higher state. As for the groups with moderate and lower levels of physical activity, they may lack exercise habits. With increasing age, their physical activity levels decline at a steady and gradual pace.

For individuals transitioning into retirement, our study identified some factors influencing changes in physical activity levels. We found having an annual income exceeding 40,000 yuan predicts membership of the “sustained low level of physical activity” trajectory when compared with the “sustained high level of physical activity with significant fluctuations”. Those in the “middle level of physical activity with gradual decline” trajectory were more likely to have an annual income exceeding 40,000 yuan, and reside in urban areas, and have a fondness for playing Mahjong when compared with the “sustained high level of physical activity with significant fluctuations”.

We observe that, regardless of the comparison between any two groups, residents with relatively higher annual incomes exceeding 40,000 yuan tend to engage in less physical activity. This finding differs from previous research results [51, 52], and it may be attributed to the context of Chinese older adults. Within the occupational milieu of China, it has been observed that as elderly individuals approach the age of retirement, higher income levels are typically indicative of their occupying more pivotal roles within the workforce. This conveys their often elevated responsibilities and corresponding higher workloads, thereby engendering augmented work-related burdens, leading to less time available for physical activity. Previous studies have shown that the rapid economic development in China over the past three decades has led to significant improvements in living standards. However, it has also resulted in a decrease in physical activity levels among older adults [53].

In comparison to individuals with high levels of physical activity, the group engaged in moderate levels of physical activity is more likely to reside in urban areas and have a hobby of playing mahjong. The living environment has a significant impact on the physical activity levels of older adults. Studies have shown that in areas with higher urbanization and greater building density, the physical activity levels of older adults tend to be lower [8, 54]. This finding aligns with the results of our study. In urban areas, where there are more buildings and fewer open spaces such as parks, there is inevitably a negative impact on the physical activity levels of older adults [53, 55].

Playing mahjong is a popular activity among Chinese residents. Older adults who have a hobby of playing mahjong often engage in the activity frequently throughout the week, and each session typically lasts for several hours. As these individuals generally do not allocate time or have a habit of exercising, their prolonged sitting posture during mahjong sessions results in lower levels of physical activity [42, 56].

Retirement represents a critical juncture in an individual’s life course, and the retirement transition period should be regarded as a vital opportunity to enhance their quality of life. Our study offers valuable insights that can contribute to the development of well-suited intervention policies aimed at fostering healthy aging among the nation’s elderly population [57]. Future research is warranted to devise health policies tailored to the needs of older individuals approaching retirement age.

### Strengths and limitations

Compared with most of the previous studies, the major strengths of our study were long follow-up with repeated measurements both before and after retirement. Our study benefited from a rich and comprehensive dataset collected over a significant period of time, including six time points. This extensive longitudinal and prospective dataset allowed us to go beyond the existing evidence and investigate the variations in physical activity during the post-retirement years, as well as the changes following the transition to retirement throughout the consecutive follow-up periods. The next strength of our study is that we employed LCGA method to explore these potential outcomes, which led to more realistic, diverse conclusions that enhance the relevance of our study. Unlike the conventional method of categorizing subjects based on cross-sectional analysis, LCGA can detect hidden clusters that exhibit comparable patterns of development throughout a given time period. This approach makes our research findings more meaningful and relevant to real-life scenarios [33].

However, several limitations are present in this study. Firstly, we relied on self-reported data pertaining to physical activity, which was acquired through participants’ recall rather than objective measurements from professional institutions, potentially introducing recall bias. Secondly, due to the specific definition of retirement, most agricultural workers were excluded from the study, leading to a sample with relatively homogeneous employment types, socioeconomic status, and other crucial factors. Thirdly, in the initial three surveys, investigator randomly selected only half of the respondents to inquire about physical activity; it was not until the final 2018 survey that all respondents were asked relevant questions. Consequently, fewer individuals fulfilled the criteria of having answered physical activity-related questions across all four surveys, the number of participants ultimately included in the study was relatively small, which might have affected the representativeness of our sample to some extent. Last but not least, due to the complex Probability Proportional to Size sampling method used in CHARLS to select survey participants, there is a possibility that the obtained results may exhibit certain biases and may not fully reflect the true situation of the entire Chinese population.

## Conclusion

In conclusion, this research indicates that the progression of physical activity among older Chinese individuals during their transition to retirement follows various distinct trajectories. These trajectories can be predicted for specific populations by factors associated with the pre-retirement phase. Furthermore, this transitional period significantly highlights the necessity of implementing targeted policies for individuals approaching retirement age in order to foster healthy aging.

## Data Availability

The datasets analyzed is open-access to request online at charls.pku.edu.cn/.

## List of Abbreviations

CNCDs: Chronic non-communicable diseases
LCGA: Latent class growth analysis
CHARLS: China Health and Retirement Longitudinal Study
GEE: Generalized estimation equation
MET: Metabolic equivalent
OR: Odds ratios
95% CI: 95% confidence intervals
